# Zusammenhänge zwischen sozialer Herkunft, Unterrichtsform und Learning Outcomes während der Pandemie

**DOI:** 10.1007/s35834-022-00355-5

**Published:** 2022-08-24

**Authors:** Ramona Obermeier, Sonja Lenz, Christoph Helm

**Affiliations:** Linz School of Education, Abteilung für Bildungsforschung, Johannes Kepler Universität Linz, Altenberger Straße 69, 4040 Linz, Oberösterreich Österreich

**Keywords:** COVID-19, Bildungsungleichheit, Lernzeit, Lernzuwachs, Belastungserleben, COVID-19, Educational inequality, Perceived stress, Study progress, Study time

## Abstract

Die COVID-19 Pandemie hat zu flächendeckenden Schulschließungen geführt, was mit massiven Veränderungen in den Lehr- und Lernprozessen sowie den Lernergebnissen der Schüler*innen verbunden war. Während erste Leistungsstudien Lerneinbußen aufgrund der Schulschließungen im Frühjahr 2020 untersuchen, liegen bisher kaum Befunde vor, die Hinweise auf zentrale Prädiktoren für die Entwicklung von Schüler*innenoutcomes (Belastungserleben, Lernzuwachs und Lernzeit) während späterer Schulschließungen liefern. Die vorliegende Studie nähert sich dieser Frage an, indem Schüler*innenoutcomes vor und während der Schulschließungen durch die Herkunft (Akademikerhaushalt, zuhause gesprochene Sprache) und die Unterrichtsform (offen vs. traditionell) vorhergesagt werden. Die vorgestellten Mehrebenen-Regressionsmodelle werden durch Theorien zur Entstehung von Bildungsungleichheit und Konzepten offenen Unterrichts motiviert. Die Ergebnisse der Mehrebenenmodelle (*N* = 1566 Schüler*innen der Sekundarstufe II) zeigen, dass das Belastungserleben der Schüler*innen während der Schulschließungen für Kinder ohne deutsche Muttersprache höher war als bei Kindern mit deutscher Muttersprache. Hinsichtlich der retrospektiven Angaben zum selbsteingeschätzten Lernzuwachs und der Lernzeit bestehen dagegen keine (über die Unterschiede in den Eingangswerten vor COVID hinausgehende) Zusammenhänge mit der Herkunft. Bezüglich der Beziehung offener Unterrichtsformen und der retrospektiv eingeschätzten Schüler*innenoutcomes zeigt sich, dass Schüler*innen aus traditionell unterrichteten Klassen während der Schulschließungen eine statistisch signifikant höhere Lernzeit angeben als Schüler*innen, die offen unterrichtet wurden. Offen unterrichtete Schüler*innen schätzen allerdings trotz geringerer Lernzeit ihren Lernzuwachs nicht geringer ein als traditionell unterrichtete Schüler*innen.

## Abstract (en)

The COVID-19 pandemic has led to widespread school closures, which have been associated with massive changes in teaching and learning and student learning outcomes. While initial performance studies have examined learning losses due to the school closures, there are few findings to date that provide evidence on key predictors of the development of other student outcomes (stress experience, learning gain, learning time) during the school closures. This study approaches these questions by retrospectively predicting student self-assessments in these student outcomes for the periods before and during school closures by social background (academic household, language spoken at home) and instructional design (open vs. traditional). The multilevel regression models presented are motivated by theories of educational inequality and concepts of open instruction. In addition, the hypotheses tested are based on recent survey studies on COVID-19 and school. Results calculated on the basis of data from *N* = 1566 upper secondary school students show higher values in the retrospectively assessed stress experience of students without German as their native language than for children with German as their native language during school closures. With regard to the retrospectively self-assessed learning gain and learning time, however, there are no correlations with social background (beyond the differences in the baseline values before COVID). With respect to the relations between open instruction and student outcomes, it appears that students from traditional instruction report statistically significant higher learning time during school closures than students in open instruction. Students in open instruction, however, do not rate their learning gain to be lower than traditionally taught students despite less learning time.

## Einleitung

Der starke Zusammenhang zwischen der Herkunft von Schüler*innen und ihren Bildungsentscheidungen lässt sich empirisch durch eine Vielzahl an Studien belegen (zusammenfassend bei Gerhartz-Reiter 2019). Der Schule wird eine kompensatorische Funktion hinsichtlich herkunftsbedingter Unterschiede zugeschrieben (z. B. Maaz et al. 2011). Durch die Schulschließungen im Rahmen der Eindämmung der COVID-19 Pandemie ab dem Frühjahr 2020 fanden nicht nur hinsichtlich der Stoffvermittlung, sondern auch bezogen auf die soziale Funktion der Schule massive Veränderungen statt (Holtgrewe et al. 2020).

Zusammenhänge der Schulschließungen mit kognitiven und affektiven Outcomes unterschiedlicher Schüler*innengruppen wurden international in vielen Studien, die in Bezug auf Lernzeit und Leistungsdifferenzen ein heterogenes Bild zeigen, untersucht (Reviews: Helm et al. 2021a; Thorn und Vincent-Lancrin 2021; König und Frey 2022). Aus Schüler*innen- (z. B. Anger und Sandner 2020) und Elternbefragungen (z. B. Wößmann et al. 2020) lassen sich hingegen keine klaren Unterschiede in der Lernzeit ableiten. Hinsichtlich des selbst- und fremdeingeschätzten Lernerfolgs weisen einige Studien (z. B. Anger und Sandner 2020; Baier und Kamenowski 2021; Vuorikari et al. 2020) auf geringere Lernoutcomes von Schüler*innen mit niedrigerem sozioökonomischem Status hin. Auch hinsichtlich der herkunftsbedingt unterschiedlichen Entwicklungen von affektiven Merkmalen (z. B. psychische Belastung) ist die Befundlage heterogen. Studien deuten auf eine geringere Motivation, ein negativeres emotionales Erleben (z. B. Baier und Kamenowski 2020) und geringere Fähigkeiten selbstgesteuert zu lernen bei sozial benachteiligten Schüler*innen hin (Holtgrewe et al. 2020; Vuorikari et al. 2020), obschon diese Aspekte seltener untersucht wurden.

Lernergebnisse von Schüler*innen sind nicht nur von ihrer Herkunft abhängig. Bisherige Studien im Kontext regulären Unterrichts legen zudem nahe, dass kognitive und affektive Outcomes bei Schüler*innen stark mit der Art der Vermittlung der Inhalte zusammenhängen (z. B. Helmke 2017). Unterricht, der Selbstbestimmung ermöglicht und das Autonomie- und Kompetenzerleben der Schüler*innen unterstützt, gilt als besonders förderlich für beide Arten von Outcomes. Insbesondere Unterrichtskonzepte, die das selbstgesteuerte Lernen in den Fokus nehmen, erscheinen für das von hoher Selbststeuerung geprägte distance learning während der Schulschließungen bedeutsam. Derartige Unterrichtskonzepte sind in der Familie der offenen Unterrichtsformen zu finden. Einschlägiger Literatur zufolge (Heirweg et al. 2019; Schoor et al. 2015) scheint kooperativer, offener Unterricht das selbstgesteuerte Lernen und die tiefenorientierte Auseinandersetzung mit Lerninhalten zu fördern. Befunde zum distance learning zeigen, dass Fähigkeiten selbstgesteuert zu lernen positiv mit dem selbst- und fremdeingeschätzten Lernzuwachs, der Motivation und dem Wohlbefinden zusammenhängen (Steinmayr et al. 2021) und legen nahe, dass Schüler*innen aus kooperativen, offen Unterrichtssettings während der Schulschließungen einen Vorteil hatten. Allerdings wurden solche Zusammenhänge unseren Recherchen nach bisher noch nicht erforscht.

Die hier vorgestellte Studie verfolgt daher zwei Ziele: (1) Aufgrund der heterogenen Befundlage zu Veränderungen von Schüleroutcomes (z. B. Helm et al. 2021a; König und Frey 2022) und zur Zunahmen von Bildungsungleichheit (z. B. Helm et al. 2021a), steht der Zusammenhang zwischen der sozialen Herkunft der Schüler*innen und die durch die Schulschließungen bedingte Veränderung des Belastungserlebens, des Lernzuwachses und der Lernzeit im Fokus. (2) Darüber hinaus wird untersucht, ob sich Schüler*innen, die kooperativ, offen unterrichtet werden, von traditionell unterrichteten Schüler*innen in ihrer Entwicklung unterscheiden. Wir nutzen dazu retrospektive Selbsteinschätzungen von Schüler*innen.

## Bildungsbeteiligung und soziale Herkunft

Internationale Leistungsstudien (z. B. PISA, PIRLS) deuten auf starke Zusammenhänge zwischen Herkunft, Bildungsbeteiligung, Leistungsfähigkeit und motivationaler Entwicklung hin (Maaz et al. 2010; Reiss et al. 2016). Eine schlechtere Ausgangslage im Bildungserwerb besteht vor allem für Kinder, deren Eltern selbst einen niedrigen Bildungsabschluss und einen Migrationshintergrund besitzen. Diese Schüler*innen haben häufig geringere formelle Bildungsabschlüsse, überfachliche Kompetenzen und sozio-emotionale Fähigkeiten (Gerhartz-Reiter 2019). Die herkunftsbedingten Disparitäten lassen sich mit Hilfe der Habitustheorie sowie der Kapitaltheorie erklären: So verfügen Schüler*innen abhängig von ihrer Herkunft über unterschiedliche normative Grundeinstellungen (Habitus), die die Bildungsentscheidungen beeinflussen. Ferner differieren sie in den Ausprägungen des sozialen, kulturellen und ökonomischen Kapitals, das in Bildung investiert werden kann (Boudon 1974; Bourdieu 2010).

Es wird diskutiert, dass Schulen für privilegiertere Schüler*innen bessere Entwicklungsmilieus als für benachteiligte Schüler*innen bieten (Downey und Condron 2016). Ursachen dafür finden sich auf unterschiedlichen Ebenen der Schule (z. B. Einzugsgebiet, Klassenzusammensetzung, Schul- und Klassenklima). Auch schulstrukturelle Merkmale (z. B. frühe Selektion) tragen zur Reproduktion von Bildungsungleichheit bei (Baumert 2006; Gerhartz-Reiter 2019; Maaz et al. 2011). Neben Aspekten auf Schulebene muss jedoch auch die Wechselbeziehung zwischen Herkunft und Nutzung von (unterrichtlichen) Lernangeboten im Sinne des Angebot-Nutzungs-Modells (Helmke 2017; Jansen et al. 2022) berücksichtigt werden. Entsprechend existieren Belege dafür, dass Schüler*innen mit stark differierenden Entwicklungsständen in die Schule eintreten und sich diese während der Schullaufbahn verschärfen (Maaz et al. 2011). Die Zeit, die außerhalb der Schule verbracht wird, scheint dies besonders zu beeinflussen (Maaz et al. 2011). Die außerschulische Umgebung kann die Verfolgung und Erreichung von Lernzielen und die Kompetenzentwicklung in der Schule unterstützen oder hemmen und trägt demnach zu unterschiedlichen Outcomes bzw. zur differenziellen Entwicklung bei (Boekaerts und Corno 2005; Heirweg et al. 2019). Schüler*innen aus Familien mit niedrigerem Sozialstatus, die häufig wenig Unterstützung durch die Eltern erfahren, verfügen über geringere Fähigkeiten selbstgesteuert zu lernen (Heirweg et al. 2019) und geringere Medienkompetenz (Beaunoyer et al. 2020). Diese Aspekte sind vor allem im distance learning von zentraler Bedeutung (Huber und Helm 2020; Steinmayr et al. 2021; Vuorikari et al. 2020).

## Bildungsungleichheit in Zeiten der COVID-19-bedingten Schulschließungen

Studien legen nahe, dass die Schule eine kompensatorische Wirkung hinsichtlich der beschriebenen Unterschiede zwischen Schüler*innen entfalten kann (Maaz et al. 2011). Bedingt durch die Schulschließungen im Zuge der COVID-19 Pandemie verbrachten Schüler*innen jedoch einen Großteil ihrer Zeit zuhause, was diese kompensatorische Wirkung beeinträchtigt haben könnte. Um diese Vermutung zu untersuchen, fokussieren wir in der vorliegenden Arbeit drei Formen der Bildungsungleichheit:

1. Herkunftsbedingte Differenzen in der erlebten *Belastung*. Schulschließungen führten zu Kontaktbeschränkungen und damit zur Reduktion von sozialen Ressourcen (z. B. Peer-Kontakte, Interaktionen mit Lehrkräften; Holtgrewe et al. 2020). Dadurch stieg die Belastung an. Aufgrund der hohen Bedeutung sozialer Medien für die Aufrechterhaltung sozialer Kontakte während der Schulschließungen war das Risiko der sozialen Isolation für benachteiligte (Schüler‑)Gruppen, die ohnehin bereits meist über geringeres Sozialkapital verfügten, erhöht (Beaunoyer et al. 2020). Darüber hinaus ist dokumentiert, dass sozial benachteiligte Kinder während der Pandemie häufiger Aggressionen im häuslichen Umfeld (Baier und Kamenowski 2021) erlebten, was zusätzlich zu einem höheren Belastungserleben geführt haben dürfte. Entsprechend prüfen wir folgende Hypothesen:
  H1a. Das Belastungserleben ist während der Schulschließungen im Vergleich zur Zeit vor der Pandemie im Durchschnitt aller Schüler*innen statistisch signifikant höher.
  H1b. Der Zuwachs im Belastungserleben fällt für sozial benachteiligte Schüler*innen statistisch signifikant höher aus.
2. Herkunftsbedingte Differenzen im (selbsteingeschätzten) *Lernzuwachs.* Im distance learning gewannen viele häusliche Lernressourcen (z. B. häusliche Ausstattung mit technischen Endgeräten, Medienkompetenz der Schüler*innen und Eltern, Lernunterstützung der Eltern und Geschwister etc.) an Bedeutung. In einer Vielzahl an Studien (siehe Helm et al. 2021b für einen Überblick) ist dokumentiert, dass sozial benachteiligte Schüler*innen in deutlich geringerem Ausmaß über diese Ressourcen verfügten. Es zeigte sich zudem, dass sozial benachteiligte Schüler*innen häufiger Schwierigkeiten beim Lernen während der Schulschließungen hatten und öfter Lehrkräfte und Klassenkamerad*innen um Hilfe baten (Holtgrewe et al. 2020). Die Fähigkeit selbstgesteuert zu lernen und die Lernmotivation dieser Schüler*innen waren ebenfalls geringer ausgeprägt (Blume et al. 2021; Steinmayr et al. 2021). Schüler*innen aus sozial benachteiligten Familien wurden durch ihre Eltern zudem in geringerem Ausmaß unterstützt (Holtgrewe et al. 2020). Empirisch deuten erste Leistungsstudien darauf hin, dass COVID-19-bedingte Lerneinbußen für sozial benachteiligte Schüler*innen deutlich wahrscheinlicher waren als für sozial privilegierte Schüler*innen (Helm et al. 2021a). Vor diesem Hintergrund prüfen wir folgende Hypothesen:
  H2a. Der Lernzuwachs wird während der Schulschließungen im Vergleich zur Zeit vor der Pandemie im Durchschnitt aller Schüler*innen subjektiv geringer eingeschätzt.
  H2b. Der Rückgang im Lernzuwachs fällt für sozial benachteiligte Schüler*innen statistisch signifikant höher aus.
3. Herkunftsbedingte Differenzen in der (selbsteingeschätzten) *Lernzeit.* Die Schulschließungen führten zur Reduktion von Lernzeiten. Geregelte Lernzeiten, in denen sichergestellt ist, dass auch in Phasen, in denen die Motivation nachlässt oder Lernschwierigkeiten bestehen, Lernziele verfolgt werden, fielen vor allem in den ersten Wochen der Schulschließungen, die durch hohe Unsicherheiten der Schüler*innen, Lehrkräfte und Eltern gekennzeichnet waren, weg. Infolgedessen wurde in Summe weniger Zeit für das Lernen aufgewendet (z. B. Wößmann et al. 2020). Betrachtet man dagegen den Anteil an *Lernzeit zuhause*, so stieg dieser während der Schulschließungen deutlich an (z. B. Helm und Postlbauer 2021). Allerdings zeigen erste Studien, dass vor allem sozial benachteiligte Schüler*innen während der Schulschließungen weniger Zeit in häusliche Lernaktivitäten investierten (z. B. Grewenig et al. 2021), was zu folgenden Hypothesen führt:
  H3a. Die Lernzeit zuhause ist während der Schulschließungen im Vergleich zu vor der Pandemie im Durchschnitt aller Schüler*innen statistisch signifikant höher.
  H3b. Der Zuwachs in der Lernzeit fällt für sozial benachteiligte Schüler*innen statistisch signifikant niedriger aus.

Zusammenfassend gehen wir davon aus, dass die Herkunft einen Prädiktor für das *Belastungserleben*, den subjektiven *Lernzuwachs* und die *Lernzeit* während der Schulschließung darstellt.

## Offener Unterricht vor und während Schulschließungen

Es kann anhand bisheriger Befunde zur Unterrichts- und Unterrichtsqualitätsforschung nicht eindeutig abgeleitet werden, ob Prozessmerkmale der Schule und des Unterrichts in der Lage sind, die bestehende Bildungsungleichheit zu kompensieren (Scheerens 2017). Dennoch wird davon ausgegangen, dass Schule Ungleichheiten der Schüler*innen entlang verschiedener Dimensionen reduzieren kann (Downey und Condron 2016). Die Individualisierung des Unterrichts wird als ein probates schulinternes Mittel angeführt (Altrichter et al. 2009; Fuhrbach 2021). Es wird angenommen, dass kooperative und offene Unterrichtskonzepte die Fähigkeit selbstgesteuert zu lernen schulen (Fischer et al. 2020; Helm 2016), was wiederum positiv mit Selbstlernphasen im distance learning zusammenhängt (Helm und Huber 2022). Ein offenes Lernformat, das in Österreich weit verbreitet ist, ist das Cooperative Open Learning (COOL). Vorrangiges Ziel von COOL ist die Förderung der Sozialkompetenz der Schüler*innen durch Unterstützung der Entwicklung von Selbstständigkeit und Verantwortung. Kernelemente sind Zeitfenster in der Stundenplanung, in denen die Schüler*innen selbst entscheiden können, welche Arbeitsaufgabe sie wann, wo und wie bearbeiten. Aufgrund dieser „offenen Unterrichtszeitfenster“ sind COOL-Schüler*innen daran gewöhnt, Aufgaben eigenständig zu bearbeiten (Helm 2016). Das COOL-Konzept legt den Schwerpunkt auf schülerzentrierte und kooperative Lernsituationen (d. h. Teamarbeit), um selbstgesteuertes Lernen (z. B. metakognitive Fähigkeiten) und soziale Fähigkeiten der Schüler*innen zu fördern (Neuhauser und Wittwer 2002). Digitale Tools (z. B. Moodle, MS Teams), die im distance learning relevant wurden, wurden in COOL-Schulen großteils bereits vor der Pandemie implementiert. Die Schüler*innen hatten demnach schon erste Erfahrungen mit diesen und konnten sie vermutlich während der Schulschließungen besser nutzen. Die Erreichbarkeit von Schüler*innen oder Lehrkräften stellte eine Herausforderung im distance learning dar (z. B. Helm et al. 2021b). Der höhere Erfahrungsschatz der Schüler*innen und Lehrkräfte und die bereits vor der Pandemie bestehende stärkere Zusammenarbeit (auch mittels digitaler Tools) im COOL-Unterricht sollte diese jedoch abgeschwächt haben. Schon vor den Schulschließungen haben COOL-Lehrkräfte den Schüler*innen ausführliche Rückmeldungen zu ihren Arbeitsaufträgen gegeben, um das Lernen der Schüler*innen individuell zu unterstützen. Vor diesem Hintergrund ist von einer möglichen förderlichen Wirkung des COOL-Unterrichts auf die Entwicklung von Schüleroutcomes während der Schulschließungen auszugehen. Hinsichtlich des Zusammenhangs zwischen der Unterrichtsform (COOL vs. traditionell), die vor den Schulschließungen eingesetzt wurde, und den Schüler*innenoutcomes im distance learning liegen bislang keine Befunde vor. Vereinzelt wurden jedoch positive Zusammenhänge zwischen offenen Unterrichtsformen und den Fähigkeiten der Schüler*innen selbstgesteuert zu lernen, gefunden (Fischer et al. 2020; Helm 2016). Das Wissen über den Einsatz digitaler Tools und Unterrichtsmethoden, die in der Zeit der Schulschließungen relevant waren, lassen ebenfalls Unterschiede annehmen, die wir mit folgenden Hypothesen prüfen:

H1c. Der Zuwachs im Belastungserleben fällt für offen unterrichtete Schüler*innen statistisch signifikant niedriger aus.

H2c. Der Rückgang im Lernzuwachs fällt für offen unterrichtete Schüler*innen statistisch signifikant niedriger aus.

H3c. Der Rückgang in der Lernzeit fällt für offen unterrichtete Schüler*innen statistisch signifikant niedriger aus.

## Methode

### Design

Im Rahmen einer vom 14. April bis 23. Juni 2021 durchgeführten Schüler*innenbefragung kam ein Onlinefragebogen zum Einsatz, welcher über Kontakte des COOL-Impulszentrums und der Abteilung für Bildungsforschung an der Johannes Kepler Universität Linz (JKU) verteilt wurde. Die Befragung wurde von den zuständigen Bildungsdirektionen der teilnehmenden österreichischen Bundesländer genehmigt. Die Teilnahme war für die Schüler*innen freiwillig und anonym. Da die Zuordnung der Schüler*innen zu den einzelnen Schulen mittels eines Codes, der lediglich den Schulen und der durchführenden Institution bekannt war, stattfand, wurden keine Namen erfasst. Personenbezogene Daten (z. B. Geschlecht, Alter) wurden anhand der Richtlinien zum Datenschutz erhoben.

### Stichprobe

Es liegen Daten von *N* = 1566 Schüler*innen (Durchschnittsalter: *M* = 16,58; *SD* = 1,30; 68,3 % weiblich, 9,9 % Migrant*innen, 41,2 % COOL-Klassen) an berufsbildenden höheren Schulen aus allen österreichischen Bundesländern vor, die im Rahmen einer größeren Studie zum Verhalten und Erleben von insgesamt *N* = 2290 Schüler*innen aller Schultypen während der Schulschließungen gewonnen wurden. Die Stichprobe wird anhand von Informationen der Statistik Austria bezüglich der Geschlechterverteilung und des Migrationshintergrundes von Schüler*innen in Sekundarschulen II gewichtet. Nach Gewichtung der Daten gehen 70,6 % weibliche Schülerinnen und 21,8 % Schüler*innen mit Migrationshintergrund in die Analysen ein. 13,3 % der Schüler*innen stammen aus Familien, in denen die Mutter über einen akademischen Abschluss verfügt. Dieser Anteil liegt etwas unter dem Schnitt der österreichischen Gesamtbevölkerung der Frauen zwischen 25 und 64 auf 17,2 % zu (Statistik Austria).

### Instrumente

Für die vorliegende Studie wurden Single-Items zu Belastungserleben („Wie hoch war deine Belastung …“), Lernzuwachs („Wie war dein Lernzuwachs …“) und Lernzeit („… zusätzlich aufgewendete Stunden für die Schule pro Woche (z. B. Lernen, Arbeitsaufträge, Hausübungen)“) eingesetzt. Die Items wurden aus der Schul-Barometer-Studie (z. B. Huber und Helm 2020) übernommen. Die erprobten Items erfassen die Schülereinschätzungen retrospektiv für die Zeit vor Corona, während der 1. Schulschließung (März, April, Mai 2020) und während der 2. und 3. Schulschließung (November 2020–Februar 2021). Die Belastung und der Lernzuwachs wurden auf einer fünfstufigen Antwortskala von 1 = niedrig bis 5 = hoch abgefragt, während die Lernzeit in Stunden/Woche erfasst wurde. Als Indikatoren für die Herkunft wurden der höchste Bildungsabschluss der Mutter (1 = akademischer Abschluss/0 = kein akademischer Abschluss) und die Zuhause gesprochene Sprache (1 = Deutsch/0 = nicht Deutsch) verwendet, die in einer Vielzahl von Studien ebenfalls herangezogen werden (z. B. Maaz et al. 2010). Die Unterrichtsform (1 = COOL/0 = traditionell) wurde mit einer dichotomen Frage erfasst. Darüber hinaus enthielt der Schülerfragebogen Fragen zum distance learning; etwa zu den Bereichen Motivation, Selbstregulationsfähigkeit und Herausforderungen während der Schulschließungen, die hier nicht weiter analysiert werden. Auch die konkrete Unterrichtsgestaltung (z. B. Medieneinsatz, Kontakthäufigkeit), die Einschätzung der Kompetenzen der Lehrkräfte (z. B. Feedback) und die konkrete Umsetzung des COOL-Unterrichts wurde aus Schüler*innenperspektive erhoben.

### Analysen

Da es sich bei den Daten um hierarchische Daten auf mehreren Ebenen (Phasen der COVID-19-Pandemie = Level 1; Schüler*innen = Level 2; Schule = Level 3) handelt, wurden Mehrebenenmodelle berechnet. Die Standardfehler wurden durch Einbezug der Schulzugehörigkeit in die Modelle korrigiert, da Unterschiede in der konkreten Ausgestaltung des distance learnings in den Einzelschulen und damit Einflüsse auf die möglichen Zusammenhänge, anzunehmen sind. Ein weiteres Spezifikum der Daten bestand darin, dass die Schüler*innen retrospektiv Angaben zum Belastungserleben, subjektiven Lernzuwachs und zur Lernzeit vor der Pandemie, während der 1. und der 2. bzw. 3. Schulschließung machen konnten. Diese retrospektiven Selbsteinschätzungen konnten durch die Umwandlung des Dateiformats als pseudolängsschnittliche Daten analysiert werden, indem die Variable Zeit als subjektive Einschätzung der Veränderung aus Retrospektive der Schüler*innen in die Modelle einfloss. Zur Frage, ob sich Schüler*innenoutcomes während der Pandemie linear entwickelten, liegen unserem Wissen nach bisher keine Studien vor. Einzig in der Schul-Barometer-Studie (Huber et al. im Druck) konnte auf Basis von retrospektiv erfassten Lehrkrafteinschätzungen ein kurvilinearer Verlauf des Lernzuwachses für die Phasen vor, während und nach der 1. Schulschließung im DACH-Raum beobachtet werden. Nachdem die aus Sicht der Lehrkräfte eingeschätzten Lernergebnisse ihrer Schüler*innen während der 1. Schulschließung stark sanken, nahmen sie danach wieder deutlich zu. Ähnliche Verläufe können auch für das Belastungserleben und für die Lernzeit angenommen werden, da sich nach der 1. Schulschließung durchaus ein gewisser Gewöhnungseffekt eingestellt haben dürfte. Aus diesem Grund werden in der vorliegenden Studie sowohl lineare als auch quadratische Verläufe getestet.

Für jeden Schüler*innenoutcome (*Belastung, subjektiver Lernerfolg, Lernzeit*) wurden separate Random-Intercept-Random-Slopes Modelle berechnet. Die Prüfung der Hypothesen, die Unterschiede in den Phasen der COVID-91-Pandemie betreffen (H1a, H2a, H3a), fand durch den Einbezug der Phase der Pandemie (Variable „Zeit“) als Prädiktor (Modelle 1) statt. Zur Prüfung des Effekts der sozialen Herkunft der Schüler*innen (H1b, H2b, H3b) wurde dieses Modell um die Prädiktoren „Sprache“ und „akademischer Abschluss der Mutter“ ergänzt (Modelle 2). Der Zusammenhang der Outcomes mit der Unterrichtsform (H1c, H2c, H3c), wurde durch Einbezug der Variable „COOL“ als Prädiktor geprüft (Modelle 3). In finalen Modellen (4) wurden simultan alle Prädiktoren aufgenommen, um die Stabilität der einzelnen Koeffizienten zu prüfen. Da die Form der Verläufe für jeden Schüler*innenoutcome ebenfalls geprüft werden sollte, wurden die finalen Modelle (4) abschließend mit quadratischen Slopes modelliert (Modelle 5).

Die Mehrebenenregressionsmodelle wurden mit der *lmer()-*Funktion im Package nlme (Version 3.1-152) unter Verwendung des restricted maximum likelihood Schätzers (REML) berechnet (Finch et al. 2014). Die Variablen wurden vorab nicht standardisiert, da es sich um dichotome Variablen handelt (Dawson 2014). Ein Modellvergleich fand mittels komparativer Fit-Indizes (AIC, BIC, log-Likelihood) und χ^2^-Differenztests statt. Auf den analysierten Outcome-Variablen sowie den Prädiktoren lag der Anteil der fehlenden Werte jeweils unter 5 %. Nach Lüdtke et al. (2007) performen bei derart wenigen Missings Imputationsverfahren nicht besser als bspw. die listwise deletion (Lüdtke et al. 2007). Auch die Betrachtung der Muster der fehlenden Werte lieferte keine Indizien für eine Systematik. Entsprechend wurden Fälle mit fehlenden Werten listenweise ausgeschlossen, da Verfahren wie Full Information Maximum Likelihood im verwendeten Package nicht implementiert sind.

## Ergebnisse

### Deskriptive Befunde

Das Belastungserleben der Schüler*innen war im Vergleich zu vor der Pandemie (*M* = 2,83, *SD* = 1,10), während der 1. Schulschließung kaum höher (*M* = 2,85, *SD* = 1,31). Für die Zeit der 2. und 3. Schulschließung berichteten die Schüler*innen einen deutlich höheren Wert (*M* = 3,83, *SD* = 1,14).

**Abb. 1:**
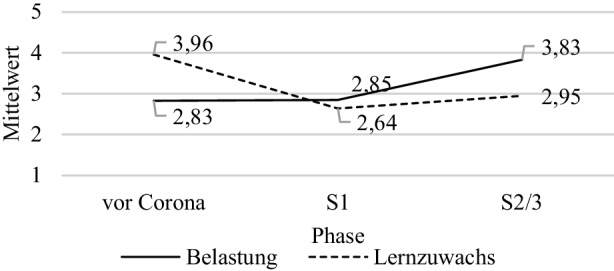
Retrospektive Schüler*inneneinschätzung des Belastungserlebens und des subjektiven Lernzuwachses zu den verschiedenen Phasen

Der selbsteingeschätzte Lernzuwachs wurde vor der Pandemie mit einem Mittelwert von *M* = 3,96 (*SD* = 0,95) im oberen Drittel der möglichen Antworten angegeben. Die Einschätzungen zur 1. Schulschließung (*M* = 2,64, *SD* = 1,16) und zur 2. und 3. Schulschließung (*M* = 2,95, *SD* = 1,19) waren erwartungsgemäß deutlich niedriger als die Eingangswerte (Abb. 1).

*t*-Tests für abhängige Stichproben zeigten, dass das Belastungserleben von Schüler*innen aus Akademikerhaushalten während der ersten Schulschließung geringer wahrgenommen wurde (Akademiker: *M* = 2,68, *SD* = 1,28; Nichtakademiker: *M* = 2,88, *SD* = 1,32; *t* (1443) = 2,03, *p* = 0,04; *d* = 0,16). Der subjektive Lernzuwachs wurde allerdings von Schüler*innen mit deutscher Muttersprache zu allen drei Phasen höher eingeschätzt (vor der Pandemie: *M*_Muttersprachler__*in_ = 4,03, *SD* = 0,88; *M*_Migrant__*in_ = 3,69, *SD* = 1,13;* t* (453,61) = −5,95, *p* < 0,01; *d* = −0,32; 1. Schulschließung: *M*_Muttersprachler__*in_ = 2,68; *SD* = 1,13; *M*_Migrant__*in_ = 2,49, *SD* = 1,25; *t* (489,59) = −2,44, *p* = 0,02; *d* = −0,15; 2./3. Schulschließung: *M*_Muttersprachler__*in_ = 3,03, *SD* = 1,16; *M*_Migrant__*in_ = 2,64, *SD* = 1,26; *t* (500,47) = −5,09, *p* < 0,01; *d* = −0,33). Das Belastungserleben der Schüler*innengruppen (traditionell vs. COOL) unterschied sich zu allen Phasen nicht statistisch signifikant. Schüler*innen im COOL-Unterricht bewerteten ihren Lernzuwachs jedoch während der 2. und 3. Schulschließung statistisch signifikant höher als Schüler*innen in traditionellem Unterricht (COOL: *M* = 3,03, *SD* = 1,15; traditionell: *M* = 2,89, *SD* = 1,21; *t* (1524) = 2,27, *p* = 0,02; *d* = 0,12).

**Abb. 2:**
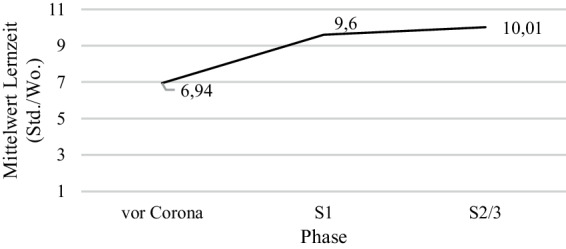
Retrospektive Schüler*inneneinschätzung des Mittelwerts der Lernzeit in Stunden/Woche zu den drei Phasen

Vor der COVID-19 Pandemie gaben die Schüler*innen an *M* = 6,94 (*SD* = 5,91) Stunden/Woche zu Hause zu lernen, wobei die Unterrichtszeit nicht berücksichtigt wurde. Die retrospektive Einschätzung der Schüler*innen während der 1. Schulschließung (*M* = 9,60; *SD* = 7,86) und der 2. und 3. Schulschließung (*M* = 10,01, *SD* = 7,40) war höher (Abb. 2).

Bezogen auf die Muttersprache und den Bildungsabschluss der Mutter stellten sich in den *t*-Tests keine statistisch signifikanten Unterschiede in der Lernzeit der Schüler*innen heraus. In traditionellem Unterricht wurde für die Phasen der Schulschließungen mehr Lernzeit angegeben als im COOL-Unterricht (1. Schulschließung: COOL. *M* = 8,99, *SD* = 7,42; traditionell: *M* = 10,05, *SD* = 7,43; *t* (1418) = −2,50, *p* = 0,01; *d* = 0,14; 2/3. Schulschließung: COOL: *M* = 9,57, *SD* = 7,31, traditionell: *M* = 10,35, *SD* = 7,46; *t* (1462) = −2,00, *p* = 0,05; *d* = 0,11).

Die Korrelationsanalysen (Tab. 1) zeigten vereinzelte Zusammenhänge der erfassten Schüler*innenoutcomes mit den Indikatoren des sozioökonomischen Hintergrunds.

**Tab. 1:** Mittelwerte, Standardabweichungen und Pearson bzw. Spearman-Korrelationen

Variable	*M*	*SD*	1	2	3	4	5	6	7	8	9	10	11
1. Sprache^a^	–	–	–	–	–	–	–	–	–	–	–	–	–
2. Hochschulabschluss Mutter	–	–	0,05^*^	–	–	–	–	–	–	–	–	–	–
3. COOL	–	–	0,04	0,01	–	–	–	–	–	–	–	–	–
4. Belastungserleben vor Corona	2,83	1,10	0,04	−0,04	−0,03	–	–	–	–	–	–	–	–
5. Belastungserleben 1. Schulschließung	2,85	1,31	−0,01	−0,05^*^	0,00	0,08^**^	–	–	–	–	–	–	–
6. Belastungserleben 2./3. Schulschließung	3,83	1,14	0,00	0,03	−0,02	−0,02	0,11^**^	–	–	–	–	–	–
7. Lernzeit vor Corona	6,94	5,91	0,03	0,01	0,02	0,20^**^	0,11^**^	0,10^**^	–	–	–	–	–
8. Lernzeit 1. Schulschließung	9,60	7,86	0,04	0,06^*^	−0,07^*^	0,04	0,16^**^	0,08^**^	0,51^**^	–	–	–	–
9. Lernzeit 2./3. Schulschließung	10,01	7,40	0,04	0,01	−0,05^*^	0,10^**^	0,07^**^	0,22^**^	0,67^**^	0,62^**^	–	–	–
10. Lernzuwachs vor Corona	3,96	0,95	0,15^**^	−0,02	−0,00	−0,16^**^	−0,02	0,06^*^	−0,05	0,04	0,05	–	–
11. Lernzuwachs 1. Schulschließung	2,64	1,16	0,07^**^	0,01	0,04	0,01	0,04	−0,13^**^	0,02	0,02	−0,01	0,09^**^	–
12. Lernzuwachs 2./3. Schulschließung	2,95	1,19	0,14^**^	0,00	0,06^*^	0,13^**^	−0,03	−0,19^**^	0,05	0,05	0,04	0,11^**^	0,30^**^

*M* Mittelwert, *SD* Standardabweichung, *COOL* Cooperative Open Learning

*p < 0,05; **p < 0,01

^a^Für dichotome Variablen wurde der Korrelationskoeffizient nach Spearman berechnet

### Mehrebenenregressionen

Die Intraklassenkorrelationen auf Ebene der Schüler*innen und Ebene der Schulen lagen bei allen Schüler*innenoutcomes über 0,05, weshalb die Berücksichtigung der hierarchischen Datenstruktur mittels Mehrebenenmodelle notwendig erscheint. Im Folgenden werden jeweils die Koeffizienten aus den Modellen mit der besten Passung (linear oder quadratisch) berichtet.

#### Belastungserleben (Tab. 2)

Das finale Random-Intercept-Random-Slope Modell mit allen Kovariaten und linearem Slope (Modell 5), zeigte einen statistisch signifikanten, linearen und positiven Zusammenhang zwischen Schulschließungen und Belastungserleben: Die Schulschließungen gingen mit höherem selbsteingeschätztem Belastungserleben der Schüler*innen einher (β = 0,59, *S.* *E.* = 0,06, *p* < 0,01). Schüler*innen, die Deutsch als Muttersprache anführten, hatten höhere Eingangswerte im Belastungserleben vor den Schulschließungen (β = 0,23; *S.* *E*. = 0,08, *p* = 0,01), gaben aber gleichzeitig für Phasen der Schulschließungen ein statistisch signifikant geringeres Belastungserleben an (Zeit * Sprache: β = −0,14, *S.* *E.* = 0,06, *p* = 0,02). Ferner berichten Schüler*innen von Müttern mit einem akademischen Abschluss von einem niedrigerem Belastungserleben vor Corona (Eingangswert) (β = −0,21, *S.* *E*. = 0,09, *p* = 0,02). Für die Einschätzung der Belastung im Verlauf der Pandemie spielte der akademische Abschluss der Mutter allerdings keine Rolle. Auch die Unterrichtsform war weder mit dem Eingangswert vor Corona noch den retrospektiven Angaben zu den Schulschließungen assoziiert.

**Tab. 2:** Mehrebenenregressionen – Belastungserleben

	Modell 1	Model 2	Model 3	Model 4	Model 5
	*B (S.* *E.)*	*B (S.* *E.)*	*B (S.* *E.)*	*B (S.* *E.)*	*B (S.* *E.)*
Intercept	2,68^***^ (0,03)	2,53^***^ (0,08)	2,71^***^ (0,04)	2,56^***^ (0,08)	2,72^***^ (0,08)
Zeit	0,50^***^ (0,02)	0,60^***^ (0,06)	0,50^***^ (0,03)	0,59^***^ (0,06)	−0,36^***^ (0,10)
Sprache	–	0,22^**^ (0,08)	–	0,23^**^ (0,08)	0,23^**^ (0,08)
Akad. Abschluss Mutter	–	−0,21^*^ (0,09)	–	−0,21^*^ (0,09)	−0,21^*^ (0,09)
Zeit * Sprache	–	−0,14^*^ (0,06)	–	−0,14^*^ (0,06)	−0,14^*^ (0,06)
Zeit * Akad. Abschluss Mutter	–	0,11 (0,07)	–	0,11 (0,07)	0,11 (0,07)
Cool Unterricht	–	–	−0,08 (0,07)	−0,09 (0,06)	−0,09 (0,06)
Zeit * Cool Unterricht	–	–	0,02 (0,05)	0,02 (0,05)	0,02 (0,05)
Zeit (quadratischer Slope)	–	–	–	–	0,48^***^ (0,04)
AIC	11.411,71	11.421,88	11.422,08	11.432,06	11.294,78
BIC	11.448,73	11.483,59	11.471,45	11.506,11	11.393,52
Log Likelihood	−5699,85	−5700,94	−5703,04	−5704,03	−5631,39
Number of observations	3537	3537	3537	3537	3537
Stichprobengröße	1179	1179	1179	1179	1179
ICC (Schüler*in)	0,00				
ICC (Schule)	0,06				
*Modellvergleiche*					
Modell 1 vs Modell 2	χ^2^ (1) = 12,58, *p* < 0,01				
Modell 2 vs. Modell 3	χ^2^ (2) = 10,37, *p* < 0,01				
Modell 3 vs. Modell 4	χ^2^ (4) = 12,78, *p* < 0,01				
Modell 4 vs. Modell 5	χ^2^ (1) = 150,04, *p* < 0,01				

*B* unstandardisierter Regressionskoeffizient*, S.* *E.* Standard Error, *AIC* Akaike-Information-Criterion, *BIC* Bayesian-Information-Criterion, *COOL* Cooperative Open Learning, *χ*^*2*^ Chi-Quadrat

^***^p < 0,001; ^**^p < 0,01; ^*^p < 0,05

#### Lernzuwachs (Tab. 3)

Die Koeffizienten im quadratischen Modell (5) deuteten auf eine geringere Einschätzung des subjektiven Lernzuwachses während der 1. Schulschließung (β = −2,27, *S.* *E*. = 0,08, *p* < 0,01) und eine Zunahme im Zeitraum der 2./3. Schulschließung (β = 0,83, *S.* *E.* = 0,03, *p* < 0,00) hin. Der Lernzuwachses vor Corona (= Intercept) war bei Schüler*innen mit Deutsch als Muttersprache höher (β = 0,27, *S.* *E.* = 0,07, *p* < 0,01). Die retrospektiven Einschätzungen der Schüler*innen über die Schulschließungen hinweg hingen dagegen weder mit dem akademischen Abschluss der Mutter noch mit der Unterrichtsform (COOL/traditionell) zusammen.

**Tab. 3:** Mehrebenenregressionen – selbsteingeschätzter Lernzuwachs

	Modell 1	Model 2	Model 3	Model 4	Model 5
	*B (S.* *E.)*	*B (S.* *E.)*	*B (S.* *E.)*	*B (S.* *E.)*	*B (S.* *E.)*
Intercept	3,72^***^ (0,03)	3,51^***^ (0,06)	3,71^***^ (0,04)	3,51^***^ (0,07)	3,78^***^ (0,07)
Zeit	−0,49^***^ (0,02)	−0,59^***^ (0,06)	−0,52^***^ (0,03)	−0,61^***^ (0,06)	−2,27^***^ (0,08)
Sprache	–	0,28^***^ (0,07)	–	0,28^***^ (0,07)	0,27^***^ (0,07)
Akad. Abschluss Mutter	–	−0,10 (0,08)	–	−0,10 (0,08)	−0,09 (0,08)
Zeit * Sprache	–	0,09 (0,06)	–	0,09 (0,06)	0,09 (0,06)
Zeit * Akad. Abschluss Mutter	–	0,05 (0,07)	–	0,05 (0,07)	0,05 (0,06)
Cool Unterricht	–	–	0,03 (0,06)	0,02 (0,06)	0,01 (0,06)
Zeit * Cool Unterricht	–	–	0,06 (0,05)	0,06 (0,05)	0,06 (0,04)
Zeit (quadratischer Slope)	–	–	–	–	0,83^***^ (0,03)
AIC	11.085,64	11.063,85	11.093,26	11.072,41	10.477,86
BIC	11.122,67	11.125,56	11.142,63	11.146,46	10.558,08
Log Likelihood	−5536,82	−5521,92	−5538,63	−5524,20	−5225,93
Number of observations	3537	3537	3537	3537	3537
Stichprobengröße	1179	1179	1179	1179	1179
ICC (Schüler*in)	0,00				
ICC (Schule)	0,08				
*Modellvergleiche*					
Modell 1 vs Modell 2	χ^2^ (4) = 36,54, *p* < 0,01				
Modell 2 vs. Modell 3	χ^2^ (2) = 31,67, *p* < 0,01				
Modell 3 vs. Modell 4	χ^2^ (4) = 35,40, *p* < 0,01				
Modell 4 vs. Modell 5	χ^2^ (5) = 578,03, *p* < 0,01				

*B* unstandardisierter Regressionskoeffizient, *S.* *E*. Standard Error, *AIC* Akaike-Information-Criterion, *BIC* Bayesian-Information-Criterion, *COOL* Cooperative Open Learning, *χ*^*2*^ Chi-Quadrat

^***^p < 0,001; ^**^p < 0,01; ^*^p < 0,05

#### Lernzeit (Tab. 4)

Die Schüler*innen gaben an, während der Schulschließungen mehr Zeit für das Lernen zuhause aufzuwenden als vor der Pandemie (positive Intercepts). Die retrospektiven Einschätzungen der Schüler*innen und die Veränderungen in diesen Einschätzungen über die Schulschließungen hinweg hingen weder mit dem Abschluss der Mutter noch mit der Muttersprache zusammen. Auch die Unterrichtsform wies keinen Zusammenhang mit der Lernzeit vor Corona auf. Allerdings deutet der negative Interaktionseffekt (Zeit * COOL: β = −0,57, *S.* *E.* = 0,19, *p* = 0,04) darauf hin, dass sich die Lernzeit in COOL-Klassen während der Pandemie stärker verringerte als in traditionellen Klassen (Tab. 4).

**Tab. 4:** Mehrebenenregressionen – Lernzeit

	Model 1	Model 2	Model 3	Model 4	Modell 5
	*B (S.* *E.)*	*B (S.* *E.)*	*B (S.* *E.)*	*B (S.* *E.)*	*B (S.* *E.)*
Intercept	7,18^***^ (0,16)	6,70^***^ (0,46)	7,24^***^ (0,21)	6,75^***^ (0,47)	6,42^***^ (0,48)
Zeit	1,70^***^ (0,10)	1,62^***^ (0,23)	1,93^***^ (0,12)	1,82^***^ (0,24)	3,80^***^ (0,39)
Sprache	–	0,54 (0,49)	–	0,55 (0,49)	0,55 (0,49)
Akad. Abschluss Mutter	–	0,01 (0,47)	–	0,02 (0,47)	0,02 (0,47)
Zeit * Sprache	–	0,09 (0,25)	–	0,13 (0,25)	0,13 (0,25)
Zeit * Akad. Abschluss Mutter	–	−0,04 (0,28)	–	−0,03 (0,28)	−0,03 (0,28)
Cool Unterricht	–	–	−0,14 (0,33)	−0,15 (0,33)	−0,15 (0,33)
Zeit * Cool Unterricht	–	–	−0,57^**^ (0,19)	−0,57^**^ (0,19)	−0,57^**^ (0,19)
Zeit (quadratischer Slope)	–	–	–	–	−0,99^***^ (0,15)
AIC	22.452,60	22.460,25	22.448,45	22.455,96	22.423,19
BIC	22.489,63	22.521,96	22.497,82	22.530,01	22.521,92
Log Likelihood	−11.220,30	−11.220,13	−11.216,22	−11.215,98	−11.195,59
Number of observations	3537	3537	3537	3537	3537
Stichprobengröße	1179	1179	1179	1179	1179
ICC (Schüler*in)	0,02				
ICC (Schule)	0,52				
*Modellvergleiche*					
Modell 1 vs Modell 2	χ^2^ (4) = 1,35, *p* = 0,85				
Modell 2 vs. Modell 3	χ^2^ (2) = 10,05, *p* < 0,01				
Modell 3 vs. Modell 4	χ^2^ (2) = 10,20, *p* < 0,01				
Modell 4 vs. Modell 5	χ^2^ (4) = 1,49, *p* = 0,83				

*B* unstandardisierter Regressionskoeffizient, *S.* *E.* Standard Error, *AIC* Akaike-Information-Criterion, *BIC* Bayesian-Information-Criterion, *COOL* Cooperative Open Learning, *χ*^*2*^ Chi-Quadrat

^***^p < 0,001; ^**^p < 0,01; ^*^p < 0,05

## Diskussion

### Zusammenfassung der Befunde

Die Befunde zeigen, dass das wahrgenommene Belastungserleben der Schüler*innen vor allem in der 2. und 3. Schulschließung erhöht war und sich gleichzeitig auch der wahrgenommene Lernzuwachs verringerte, während die selbsteingeschätzte Lernzeit zunahm. Entsprechend können die Hypothesen 1a und 2a anhand der vorliegenden Befunde bestätigt werden, da die Schüler*innen retrospektiv in den Schulschließungen höherer Belastungen und geringere subjektive Lernzuwächse angaben. Für H2a ist anzumerken, dass sich bzgl. des selbsteingeschätzten Lernzuwachses ein kurvilinearer Zusammenhang beobachten lässt. Die Schüler*innen scheinen den Lernzuwachs demnach retrospektiv in der 2./3. Schulschließung subjektiv höher einzuschätzen als zuvor. Allerdings erreicht diese Einschätzung nicht den Wert, der gemäß retrospektiven Schüler*innenangaben vor der Pandemie bestand. Die Beobachtung des kurvilinearer Zusammenhangs deckt sich mit Befunden aus der Schul-Barometer-Studie (Huber et al. im Druck), in der auf Basis von retrospektiv erfassten Lehrkräfteeinschätzungen eine sehr ähnliche Entwicklung für die Phasen vor, während und nach der 1. Schulschließung im DACH-Raum gezeigt werden konnte. Die H3a zur Erhöhung der Lernzeit wird anhand der Befunde ebenfalls bestätigt, da die Lernzeit zuhause in der Tendenz während der Schulschließungen als höher angegeben wurde als vor der Pandemie.

Bezogen auf den Einfluss der sozialen Herkunft auf die retrospektiv eingeschätzte Entwicklung des Belastungserlebens (H1b) lässt sich festhalten, dass Schüler*innen mit Migrationshintergrund zwar geringere Eingangswerte im Belastungserleben aufwiesen, aber retrospektiv ein höheres Belastungserleben während der Schulschließungen berichteten. Ein akademischer Bildungsabschluss der Mutter hing wiederum mit einem geringerem Belastungserleben der Schüler*innen vor der Pandemie zusammen, was ggf. auf bessere Unterstützungsleistungen zu Hause zurückgeführt werden könnte. Bezüglich der Veränderungen im retrospektiv eingeschätzten Lernzuwachs (H2b) und der zuhause aufgewendeten Lernzeit (H3b) fanden sich in unserer Studie keine Diskrepanzen zwischen Schüler*innen unterschiedlicher Herkunft. Damit kann Hypothese 1b teilweise bestätigt werden; die Hypothesen 2b und 3b müssen jedoch verworfen werden.

Bezüglich der Effekte des vor der Pandemie im Unterricht eingesetzten Lehrkonzeptes (COOL/traditionell) zeigten die Analysen, dass sich offen unterrichtete Schüler*innen während der Schulschließungen hinsichtlich der retrospektiv eingeschätzten Belastung und des retrospektiv eingeschätzten Lernzuwachses nicht anderes entwickelten als Schüler*innen, die vor Corona traditionell unterrichtet wurden. Die Hypothesen 3a und 3b müssen daher verworfen werden. Offen unterrichtete Schüler*innen berichten allerdings von einem stärkeren Rückgang der Lernzeit während der Phasen der Schulschließungen als Schüler*innen im traditionellen Unterricht. Hypothese 3c, die von einem Anstieg ausging, muss daher ebenfalls verworfen werden. Studien zu effektiver Lernzeit zeigen, dass nicht die Quantität, sondern die Qualität des Lernens entscheidend für den Lernzuwachs ist (Helmke 2007; Lipowski 2006; Seidel und Shavelson 2007). Schüler*innen in COOL-Klassen, könnten also über bessere Fähigkeiten selbstgesteuert zu lernen verfügen und daher im distance learning einen Vorteil haben. Ein Argument, das für diese Interpretation spricht, besteht auch darin, dass der Lernzuwachs der Schüler*innen in COOL-Klassen trotz geringerem Zeitaufwand subjektiv nicht statistisch signifikant niedriger eingeschätzt wurde. Dennoch sollte dieser Zusammenhang in Folgestudien genauer geprüft werden.

### Limitationen

Hinsichtlich des Fragebogens muss limitierend angemerkt werden, dass die Schüler*innen retrospektiv zu ihren Wahrnehmungen im distance learning befragt wurden. Die zeitliche Entfernung der Befragung von den Pandemie-Phasen könnte die Einschätzungen verfälscht haben. Entsprechend handelt es sich bei den Daten auch nicht um echte Längsschnittdaten.

Zudem ist einschränkend anzumerken, dass die Schüler*innenoutcomes und die Unterrichtsform vor der Pandemie mit Single-Items erfasst wurden und entsprechend keine Reliabilitäten berichtet werden können. Die Items wurden allerdings im Rahmen der Schul-Barometer-Studie (Helm und Huber 2022) erprobt und zeigten sich sensitive gegenüber den Schulschließungen (sowohl über die Zeit und die DACH-Länder hinweg). Gerade bezogen auf das Belastungserleben ist jedoch anzunehmen, dass sich das Konstrukt aus mehreren Facetten zusammensetzt und multikausal bzw. durch Kumulation von Stressoren entsteht. Korrelationsanalysen zeigen Zusammenhänge der Belastung mit verschiedenen Aspekten des Unterrichts und der Herkunft. In Folgestudien sollte das Belastungserleben daher differenzierter über mehrere Items und Dimensionen analysiert werden, um auch den Messfehler sowie Subdimensionen berücksichtigen zu können.

Darüber hinaus sind die subjektiven Einschätzungen zum Lernzuwachs kritisch zu betrachten, da sie nicht zwingend den wahren Lernzuwachs abbilden (Hansford und Hattie 1982). Dennoch liefern die vorgelegten Befunde bislang fehlende Einblicke in aus Schülersicht retrospektiv wahrgenommene Veränderungen ihrer Outcomes zeigen, inwiefern diese Entwicklungen von zentralen Einflussgrößen (Herkunft, Unterrichtsform) abhängen.

Neben der Sprache und dem Bildungsabschluss der Mutter wäre in nachfolgenden Untersuchungen die Analyse weiterer Hintergrundmerkmale (z. B. kulturelle Identität, elterliche Aspirationen) gewinnbringend. Zwar liegen im Datensatz Informationen zur technischen Ausstattung zu Hause und auch zur Unterstützungsleistung der Eltern im distance learning vor, allerdings korrelierten diese nicht mit den Outcome-Variablen und wurden daher nicht untersucht.

Mit Blick auf die Stichprobe ist positiv hervorzuheben, dass es sich um eine große Stichprobe handelt, die durch Gewichtung der Merkmale Geschlecht und Migrationshintergrund an die Grundgesamtheit angeglichen wurde. Trotz der vorgenommenen Gewichtung, besteht dennoch die Möglichkeit einer Verzerrung der Befunde, da weitere Gewichtungsfaktoren (z. B. Bildungshintergrund der Eltern) nicht berücksichtigt werden konnten. Darüber hinaus handelte es sich bei den befragten Schüler*innen um Sekundarstufenschüler*innen, die berufsbildende Schulen besuchten. Es kann angenommen werden, dass der Einfluss der Eltern und damit der Herkunft im Bildungsverlauf tendenziell abnimmt, da in der Adoleszenz die Peer-Gruppe zunehmend an Bedeutung gewinnt und auch die Fähigkeiten der Selbstregulation zunehmen (Steinmayr et al. 2021). Effekte der Herkunft könnten daher bedingt durch das fortgeschrittene Alter der Schüler*innen in der vorliegenden Stichprobe ausbleiben. Die Befunde lassen sich damit nicht auf andere Schulstufen übertragen.

Schließlich konnte aus Anonymitätsgründen die Klassenzugehörigkeit der Schüler*innen nicht erfasst werden, sodass in der vorliegenden Studie zwar für die Schulzugehörigkeit nicht aber für die Klassenzugehörigkeit kontrolliert werden konnte. Auch eine Differenzierung hinsichtlich der besuchten Schulart war aufgrund der Form der Abfrage nicht möglich.

### Implikationen

Die Schulschließungen führten zu einem Anstieg im Belastungserleben der Schüler*innen (z. B. COPSY-Studie Kaman et al. 2021). Dabei sind unterschiedliche Arten von Belastung zu differenzieren: Im Zusammenhang mit der Pandemie und den Schulschließungen werden insbesondere Ängste vor Krankheit und vor Impfung, fehlende soziale Kontakte (die zu Einsamkeit und Traurigkeit führen); Herausforderungen im Homeschooling durch fehlende Infrastruktur und Unterstützung, Angst vor Arbeitsplatzverlust der Eltern und drohender Armut, finanzielle Sorgen, unsichere Zukunftsperspektiven, (familiärer) Stress und Konflikte, Unruhe, Schlaf- und Essstörungen bis hin zu posttraumatische Symptomen (Berghammer 2020; Brooks et al. 2020; Budimir et al. 2021; Culen 2022; Schabus und Eigl 2021; Zartler et al. 2022) identifiziert.

Einschlägige Studien haben gezeigt, dass viele Schüler*innen mit dem Distance Learning (z. B. Holtgrewe et al. 2020; Müller 2020) und der sozialen Isolation (z. B. Letzel et al. 2020; Lochner 2020) überfordert sind (z. B. Langmeyer et al. 2020). Darüber hinaus fühlen sich die Schüler*innen allgemein (z. B. Huber et al. 2020; Schreiner et al. 2020) und psychisch belastet (z. B. Wößmann et al. 2020, 2021), was sich in einem häufigeren Auftreten von psychischen Auffälligkeiten und Symptomen wie Angst und Depression zeigt (Kaman et al. 2021; Schlack et al. 2020). Studien zeigen zudem, dass sozial schlechter gestellte Schüler*innen über eine deutlich höhere Belastung berichten als sozial besser gestellt Schüler*innen (z. B. Baier und Kamenowski 2020; Holtgrewe et al. 2020).

Da das selbsteingeschätzte Belastungserleben der Schüler*innen während der Schulschließungen auch in der durchgeführten Studie nachweislich höher war als vor Beginn der Pandemie, ist es nötig über Maßnahmen nachzudenken, die die erlebte Belastung reduzieren. Im Fokus solcher Angebote sollten Workshops zur Vermittlung von Coping Strategien, Stressbewältigung oder zum Aufbau von sozio-emotionalen Kompetenzen stehen (Hadar et al. 2020). Auch die Unterstützung beim Aufbau von Selbstlernfähigkeiten ist zentral (Räisänen et al. 2020), da durch hohe Fähigkeiten selbstgesteuerten Lernens zusätzlich positiv Einfluss auf die Kompetenz- und Selbstwirksamkeitserwartungen der Heranwachsenden genommen werden kann. Studien (z. B. Räisänen et al. 2020) zeigen, dass die soziale Isolation im Zuge der Kontaktbeschränkungen für verschiedene Personengruppen eine besonders große Herausforderung darstellte. Soziale Kontakte sind eine wichtige Ressource im Umgang mit Stress (Beaunoyer et al. 2020). Die Ermöglichung der sozialen Vernetzung sowie das Bereitstellen von Austauschplattformen und die Förderung des Gemeinschaftsgefühls sind daher auch im distance learning zu forcieren. Wichtig wäre über alternative, sichere Kontaktmöglichkeiten für Kinder und Jugendliche bei künftigen Schulschließungen nachzudenken: Könnte z. B. das in Österreich sehr stark ausgeprägte Vereinswesen mit Outdoor-Aktivitäten (für geimpfte und getestete Kinder) ähnlich den bisher in den Sommerferien angebotenen „Ferien-schecks“ Kontaktmöglichkeiten eröffnen?

Die durchgeführte Studie deutet auf eine Zunahme der Lernzeit hin, was auf eine zunehmende Anpassung an das distance learning schließen lässt. Nachdem die Umstellung während des ersten Schulschließungen sehr abrupt stattfand und die Schüler*innen zu Beginn nur spärlich mit Aufgaben versorgt wurden, könnte die Zunahme auf einen reibungsloseren Ablauf des distance learnings während der zweiten und dritten Schulschließung hinweisen. Allerdings muss aufgrund der hohen Varianz innerhalb der Lernzeit der Schüler*innen davon ausgegangen werden, dass das nicht auf alle gleichermaßen zutrifft. Der beobachtete Anstieg sollte somit nicht darüber hinwegtäuschen, dass für einzelne Schüler*innen höherer Unterstützungsbedarf besteht, der z. B. durch remediale Maßnahmen (Sommerschule, Förderunterricht etc.) adressiert werden muss. Andererseits steht ein hoher Umfang an Lernzeit nicht per se mit hohem Lernzuwachs in Beziehung (z. B. Dettmers et al. 2010). Auch im Kontext der COVID-19 Pandemie zeigt sich, dass hohe Lernzeit negativ mit der gelingenden Bewältigung des distance learnings zusammenhängt (Züchner und Jäkel 2021). Um dieser Entwicklung entgegenzutreten, ist die Förderung von Lernstrategien sowie die Unterstützung der Schüler*innen hinsichtlich effektiver Lernzeitnutzung zentral.

Die Studie liefert einen ersten Überblick über die retrospektiv eingeschätzte Veränderung des subjektiv wahrgenommenen Belastungserlebens, Lernzuwachses und der Lernzeit. Sie zeigt ferner Zusammenhänge zwischen der sozialen Herkunft und diesen Schüleroutcomes auf. Da sich vor allem die zuhause gesprochene Sprache der Schüler*innen als prädiktiv erwiesen hat, wäre es besonders wichtig, der Gruppe von Schüler*innen mit Migrationshintergrund den Zugang zur Notbetreuung und zu remedialen Maßnahmen zu ermöglichen und gezielte Unterstützungsangebote für diese zu entwickeln.

Die Analyse verdeutlicht allerdings auch, dass die Unterrichtsform vor der Pandemie das Verhalten und Erleben der Schüler*innen während des Distanzunterrichts beeinflusste. Kooperativer, offener Unterricht gemäß des COOL-Konzepts könnte potenziell förderlich sein, da die Schüler*innen in COOL-Klassen trotz geringerer Lernzeiten über keine höheren selbsteingeschätzten Lerneinbußen berichteten. Diese Vermutung braucht weiterführende Forschung, da durchaus denkbar ist, dass ein positiver Effekt des offenen Unterrichts nicht für alle Schülergruppen gilt; insbesondere nicht für bildungsferne Schüler*innen. Da die Fähigkeiten zum selbstgesteuerten Lernen häufig mit der sozialen Herkunft der Schüler*innen assoziiert sind (Holtgrewe et al. 2020; Vuorikari et al. 2020), könnten gerade bildungsfernere Schüler*innen im COOL-Unterricht benachteiligt sein.
